# Application of Decision-Analytic Models to Evaluate Integrated Care Interventions for Cardiometabolic Multimorbidity: A Systematic Review

**DOI:** 10.5334/ijic.9075

**Published:** 2026-04-27

**Authors:** Elvis Omondi Achach Wambiya, Duncan Gillespie, Robert Akparibo, James Odhiambo Oguta, Catherine Akoth, Peter Otieno, Peter J. Dodd

**Affiliations:** 1 Sheffield Centre for Health and Related Research (SCHARR), School of Medicine and Population Health, University of Sheffield, Sheffield, United Kingdom; 2 Chronic disease management (CDM) unit, African Population and Health Research Center (APHRC), Nairobi, Kenya

**Keywords:** decision-analytic models, integrated care, multimorbidity, cardiometabolic diseases

## Abstract

**Introduction:**

Integrated care is increasingly adopted to address the needs of patients with multimorbidity, but cost-effective configuration of integrated healthcare pathways remains unclear. This study reviewed decision analytic models (DAMs) used in economic evaluations of integrated care interventions for cardiometabolic multimorbidity.

**Methods:**

A systematic search of eight electronic databases was conducted to identify peer-reviewed articles published in English until November 2024. Studies using DAMs to evaluate integrated care interventions for patients at risk or having cardiometabolic multimorbidity were included. Data on DAMs characteristics, integrated care models evaluated, and diseases were summarised. The quality of reporting was assessed using Philips (2006) checklist.

**Results:**

Sixteen studies met inclusion criteria. Most studies (81%) were cost utility analyses, focused on hypertension and/or diabetes concordant multimorbidity (69%). High-income countries accounted for 69% of the studies. Markov models were used the most (63%), with only three studies employing individual patient simulation (microsimulation) models. Few studies were explicit about data validation and reporting uncertainty.

**Conclusion:**

Economic evaluations of integrated care cardiometabolic multimorbidity should adopt microsimulation to better capture patient-level interactions and health outcomes. Better reporting of validation and uncertainty is needed. There is limited application of DAM-based economic evaluations of integrated care in low- and middle-income countries.

## Introduction

Chronic diseases are generally defined as conditions lasting at least a year and requiring continued medical attention or limiting activities of daily living or both [1]. They are a leading cause of morbidity, mortality, and disability globally, making them an important focus for health systems [2,3,4,5]. Recent evidence highlights an increasing global burden of chronic diseases which is attributable to socio-demographic and lifestyle changes, and increased life expectancy due to improved therapies [6,7,8]. This increase has contributed to the growing number of people living with multiple chronic conditions making multimorbidity, the simultaneous existence of two or more chronic diseases in an individual, a pertinent public health topic [9,10,11]. Multimorbidity is associated with increased disability, morbidity and mortality, reduced quality of life, and polypharmacy leading to adverse drug reactions [12,13,14]. In addition, it results in higher health care costs to the patients affected and the health system [15,16,17]. Given the complex array of different types of multimorbidity, the contexts of the individuals’ lives and the services that are involved in treating it, understanding how to cost-effectively improve services that treat multimorbid patients is a major challenge for health systems.

Multimorbidity carries a significant burden globally and the distribution and patterns vary across populations, geographical areas, and health care settings [18,19]. A recent meta-analysis of 68 community-based studies among people aged 45 years and above estimated that at least a third of these populations have two or more chronic diseases with the prevalence being higher in high-income countries (HICs) than in low- and middle-income countries (LMICs) [20]. Cardiometabolic multimorbidity is considered one of the most common types of multimorbidity [21,22,23]. In LMICs however, there is a rising burden of multimorbidity linked to the changing disease landscape characterised by a rise in chronic non-communicable disease (NCD) burden in the context of persistent chronic communicable diseases [21,24]. This inevitably puts pressure on the health systems of these countries which have been primarily designed to address acute episodic care. This consequently leads to fragmentation of health services, yet patients with multimorbidity have higher utilisation and complex needs for the comorbidities and their complications [16,25,26]. Due to the complex nature of multimorbidity, a more comprehensive approach to service delivery that transcends beyond a single-disease focus and is person-centred is needed. However, this would likely require large scale system change to services that would in turn require careful consideration of the relative cost-effectiveness of the alternative options.

Integrated care has been widely adopted to reduce fragmentation and promote comprehensive delivery and promote efficiency of health services [27]. Traditionally more prominent in HICs, integrated care is increasingly gaining prominence in LMICs to address unique health challenges faced by people with multiple chronic diseases [28,29,30]. Different integrated care models have been developed for health care delivery in diverse service contexts to meet the needs of patients with multimorbidity [31,32,33]. Existing evidence, mainly from trial settings, demonstrates the effectiveness of integrated care in improving access to and utilisation of care, quality of care, service delivery, clinical outcomes, and cost-saving for people with multimorbidity [34,35,36]. However, the diversity of integrated care models and limited evidence from real-world studies presents a challenge for economic evaluations aimed at decision-making on integrated care and indicates the need for model-based appraisals of alternative options for integrated care tailored to specific service and population contexts.

Decision analytic models (DAMs) provide a systematic approach to evaluate the impact of health interventions on costs and outcomes under alternate scenarios [37]. They use mathematical relationships to define a series of possible consequences that would occur from a set of alternatives being evaluated, and can be implemented through different model-based approaches [38,39,40]. DAMs are particularly suited to addressing the decision-making challenges in integrated care as they enable the flexible specification of the population, disease mechanisms and diverse intervention components, allowing the computation of cost-effectiveness metrics that allow comparisons of different specifications of integrated care in the context of multimorbidity. Although some studies have been published using DAMs to model the impact of integrated care for people with multiple diseases in diverse settings [41,42,43], there has been no attempt at synthesising the modelling approaches taken in a systematic review to understand and appreciate the breadth and quality of evidence. Therefore, the present systematic review aimed to answer the question, how have DAMs been applied to evaluate the health economic impact of integrated care models for patients with cardiometabolic multimorbidity? The aim was to assess the suitability of the DAMs found for assessing the cost-effectiveness of integrated care in the context of cardiometabolic multimorbidity. Based on the review results, we provide recommendations for best practice in decision-analytic modelling to inform decision making regarding integrated care models for people with cardiometabolic multimorbidity.

## Methods

The systematic review followed methods specified in a registered protocol on PROSPERO (CRD42023407278) [44]. The findings of this systematic review are reported following the Preferred Reporting Items for Systematic Reviews and Meta-Analysis (PRISMA) guidelines [45,46].

### Search strategy and literature search

A systematic literature search was conducted in eight electronic peer-reviewed databases including Medline, Web of Science, EMBASE, Cumulative Index to Nursing and Allied Health Literature (CINAHL), APA Psychinfo, Econlit, Scopus, and the Cochrane register of controlled trials between 20/11/2023 and 15/12/2023 and updated in December 2024 and June 2025, in English language, without limits in the time frame. The search strategy captured four key concepts: 1) model-based health economic evaluations, 2) integrated care, 3) chronic diseases, and 4) cardiometabolic diseases. The search strategy was initially piloted in Medline, Embase and Web of Science, and was refined with the help of an information specialist and adapted for each specific database. Full search terms are available in supplementary note 1.

### Inclusion criteria

Eligible studies included economic evaluations reporting cost-effectiveness or cost-utility (where the outcomes are measured in quality-adjusted life years) outcomes [47,48]. Descriptive studies, opinion pieces, conference or dissertation abstracts and protocols were excluded. We defined cardiometabolic multimorbidity as the existence of two or more chronic diseases in the same individual, at least one of which was a cardiometabolic disease. Concordant multimorbidity is defined as the co-existence of two or more chronic diseases all of which are cardiometabolic diseases, while discordant cardiometabolic multimorbidity is existence of two or more chronic diseases at least one of which is a cardiometabolic [49,50]. Integrated care was defined as health service delivery containing two or more components of the chronic care model (CCM) [51,52], and at least one element of Singer et al.’s, (2011) [53] framework for measuring integrated patient care for patients with multiple or complex chronic conditions. Studies not published in the English language, and review papers were excluded. We checked through the reference list of review papers to identify potentially relevant studies that met our inclusion criteria. The inclusion and exclusion criteria summarised using the population, intervention, comparator, outcome (PICO) framework are presented in supplementary table 2.

### Study selection

The studies identified by the searches were independently screened by three reviewers. Using a predefined selection checklist, the reviewers (EW, JOO, and CA) first screened the titles and abstracts and those that met the eligibility criteria proceeded to full text screening. Reviewers were blind to each other’s decisions throughout the screening process and any conflicts identified from the screening were resolved through discussion with a fourth reviewer (either RA, DG, or PD). Endnote software was used for the removal of duplicates while Covidence software aided the screening. A detailed explanation of the process is provided in supplementary note 2.

### Data extraction

Data was extracted electronically by two reviewers (EW and JOO) using a pre-specified Microsoft Excel spreadsheet. The data extraction tool was piloted to ensure that it captured all the required information based on the review objectives. The data that was extracted from the selected studies pertained to study characteristics (study title, authors, year of publication, study setting, study aim, target population), details regarding the decision-analytic model (model type/approach, integrated care model/intervention evaluated, comparators, model assumptions, model inputs and their sources, multimorbidity conditions modelled, disease parameters included, model limitations), results and conclusions of the study. The findings are reported on a summary table and further described narratively.

### Quality assessment

We used the Philips et al., (2006) checklist to evaluate the quality of the included studies in three domains: structure, data inputs, and consistency [54]. The assessment was completed by one reviewer (EW) and validated by at least one of the co-authors (JOO, DG, RA, or PD). For each item on the checklist, a value of “yes,” “no”, “unclear” or “not applicable” was attributed, which corresponded to numeric values of 1, 0, and 0.5 respectively. We then calculated a mean quality score for each study and for each item across the studies.

### Data synthesis

We undertook a narrative synthesis of the data to summarise and appraise the identified model-based economic evaluations of integrated care for cardiometabolic multimorbidity. We first summarised the population and integrated interventions modelled by the geographical distribution, integrated care model types, and disease conditions modelled. Secondly, we described the decision-analytic approaches used including the type of DAMs, model perspectives, model horizon, and model adaptations which are important aspects in the modelling of integrated care. We used the taxonomy of health economic model structures by Brennan et al. (2006) to categorise the models developed in the selected articles [39] (Table 1). Finally, we appraised the quality of the DAMs used for the economic evaluation of integrated care using the Philips (2006) checklist [54]. This process enabled the critical synthesis of the considerations in the use of DAMs in economic evaluations of integrated care for cardiometabolic multimorbidity. Definitions of terms used for this review are outlined in supplementary table 1. We have presented results of the economic evaluations which may be useful for readers in supplementary note 3 and supplementary table 6.

**Table 1:** Taxonomy of model structures for economic evaluation.


			A	B	C	D

			COHORT/AGGREGATE LEVEL/COUNTS		INDIVIDUAL LEVEL	
	
			EXPECTED VALUE, CONTINUOUS STATE, DETERMINISTIC	MARKOVIAN, DISCRETE STATE, STOCHASTIC	MARKOVIAN, DISCRETE STATE, INDIVIDUALS	NON-MARKOVIAN, DISCRETE-STATE, INDIVIDUALS

1	No interaction allowed	Untimed	Decision tree rollback	Simulated decision tree (SDT)	Individual sampling model (ISM): Simulated patient-level decision tree (SPLDT)	
	
2		Timed	Markov model (evaluateddeterministically)	Simulated Markov model (SMM)	Individual sampling model (ISM): Simulated patient-level Markov model (SPLMM) (variations as in quadrant below for patient level models with interaction)	

3	Interaction allowed	Discrete time	System dynamics (finite difference equations, FDE)	Discrete time Markov chain model (DTMC)	Discrete-time individual event history model (DT, IEH)	Discrete individual simulation (DT, DES)
	
4		Continuous time	System dynamics (ordinary differential equations, ODE)	Continuous time Markov chain model (CTMC)	Continuous time individual event history model (CT, IEH)	Discrete event simulation (CT, DES)


*Source*: Adopted from Brennan et al. (2006).

## Results

**Figure 1:**
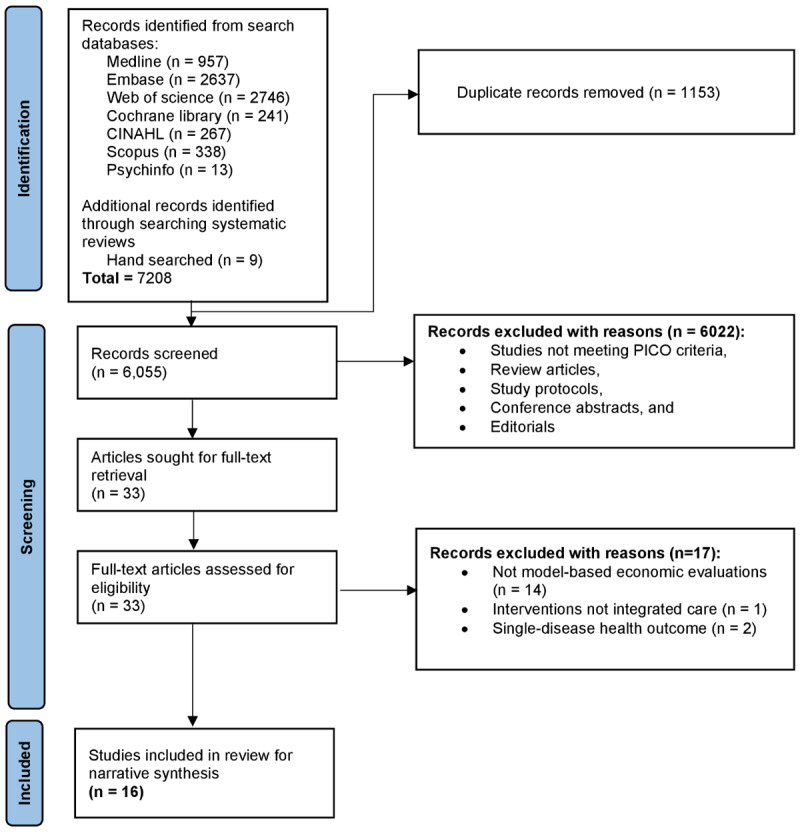
PRISMA flow chart of identification, screening, and final inclusion of articles.

The results of the study selection are presented in the PRISMA flow chart (Figure 1). We ultimately included 16 articles in the study for synthesis.

### Populations and interventions

#### Settings and target populations

**Figure 2:**
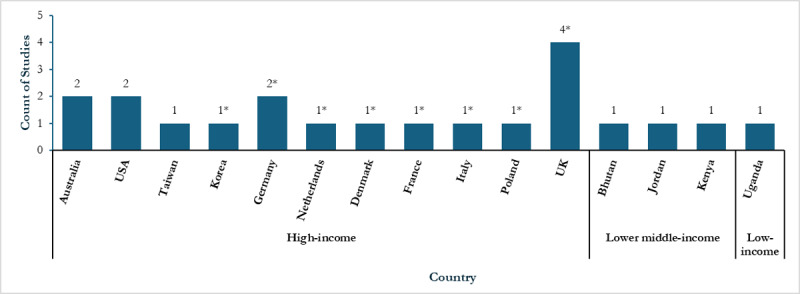
Geographical distribution of included studies. *Countries contained in one multicountry study, USA: United States of America, UK: United Kingdom.

Eleven studies were conducted in high income countries, three in lower middle-income countries (Bhutan [55], Jordan [56], and Kenya [57]), and one in a low-income country (Uganda [41]): see Figure 2. Modelled populations ranged between 15 and 75 years of age. In nine of the included articles, the starting population were either previously diagnosed with or currently had hypertension [58,59,60], type 2 diabetes mellitus [42,43,56,61], HIV [41], multiple disease risk factors [55], or a combination of these [43]. Seven studies had a baseline population starting without disease [57,62,63,64,65,66,67].

#### Disease conditions modelled

The review focused on both concordant and discordant cardiometabolic multimorbidity. Most of the included studies involved concordant multimorbidity where the modelled diseases share similar underlying causes, risk factors, and management. Diabetes was the primary disease in eight of the 16 included studies [42,43,55,56,61,63,64,66], hypertension the primary disease in four of the studies [55,58,59,60], HIV was the primary disease modelled in two studies conducted in SSA [41,57], while atrial fibrillation, cancer, and chronic kidney disease (CKD) were the primary disease in one study each [62,67] (Supplementary table 3).

**Figure 3:**
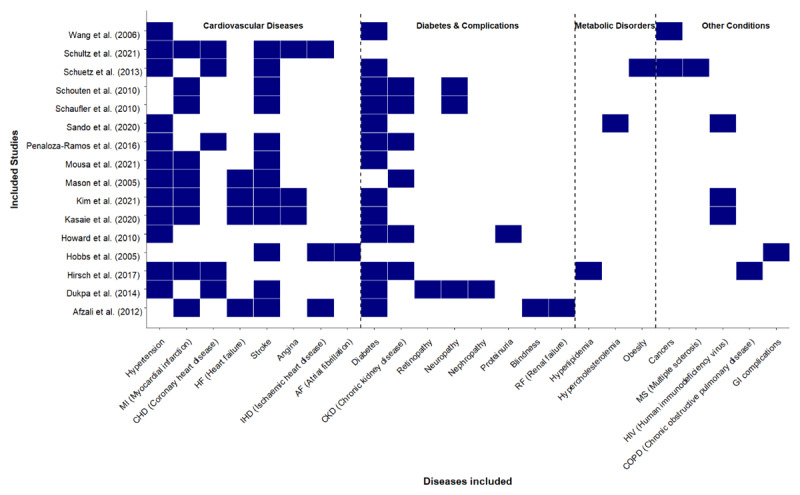
Patterns of multimorbidity modelled in selected studies.

Figure 3 shows patterns of disease conditions modelled in the selected studies. The most frequent disease pattern modelled in the studies featured hypertension and/or diabetes as index conditions paired with their related cardiovascular and metabolic complications. Two studies modelled HTN as the primary disease and related complications [59,60]. Five studies modelled HTN/T2DM comorbidity with T2DM as the primary disease with the other diseases being CVD and T2DM-related complications [55,56,61,64,68]. Two studies modelled HTN/T2DM comorbidity and also included metabolic diseases (hyperlipidemia, metabolic syndrome, obesity) and other diseases (COPD, cancers) with T2DM as the main disease [43,63,66]. One study modelled HTN/T2DM comorbidity with HTN as the primary disease [58]. Two studies conducted in SSA modelled HIV as the primary disease and HTN, T2DM, and metabolic diseases as the other conditions [41,57]. One study modelled atrial fibrillation as the primary disease and CVD and gastrointestinal complications as the other diseases [62]. Chronic kidney disease was modelled as the primary disease alongside HTN and T2DM in one study [65]. Cancer was the primary disease modelled alongside HTN and T2DM in another study [67] (Figure 3).

#### Interventions modelled

The integrated care models or interventions evaluated varied from health care provider-led interventions (e.g., pharmacist-led), integrated screening and treatment, comprehensive disease management programs, and a quality improvement intervention. The details and characterisation of the integrated care interventions are presented in Table 1 and supplementary table 4, respectively. There was a high level of diversity in the interventions modelled under the definition of integrated care in the selected studies. Two studies conducted in Jordan [56] and USA [60] evaluated pharmacist-led care and medication therapy management (MTM) for diabetes and hypertension respectively. Hirsch et al., [43] evaluated collaborative endocrinologist-pharmacist intense medication management for diabetes in the USA. One study in Australia [42] evaluated high level patient nurse involvement, while another study in the UK [63] focused on specialist nurse-led clinics for diabetes. Integrated screening and treatment constituted most of the included studies. The study conducted in Bhutan [55] and a multicountry study in six European countries [66] evaluated integrated screening, treatment and management for diabetes and hypertension among other related risk factors. A study in Uganda [41] evaluated integrated screening and treatment of NCDs into HIV care. Early detection and secondary prevention of diabetes was evaluated in one study in Germany [64] while a study in Australia [65] evaluated primary care-based screening for chronic kidney disease risk factors and improved management. Community-based integrated screening was evaluated in two studies in Kenya [57] and Taiwan [67]. One UK study examined whole population screening and opportunistic screening compared to usual care [62]. Self-monitoring and management of blood pressure in hypertensive patients was evaluated in one UK study [58] while another study conducted in the Netherlands [61] evaluated a quality improvement collaborative for patients with T2DM.

### Analytic approaches used

This section provides details of the DAMs developed in the selected studies. Characteristics of the DAMs are summarised in supplementary table 5.

#### Economic approach

Cost utility analysis (CUA) was used by 13 studies [41,43,55,57,58,59,60,61,63,64,65,66,68], while three studies [56,62,67] were cost-effectiveness analysis studies whose outcomes were not based on utilities (Supplementary table 3).

#### Discount rates

Discount rates used in the selected studies varied among countries due to the conventions used in different countries. The discount rates for costs ranged between 3% and 5% while the discount rates for outcomes ranged between 1.5% and 5%.

#### Model type

Of the 16 selected economic evaluations, nine used simulated Markov models to evaluate the impact of the integrated care interventions [42,56,58,59,61,63,64,67]. Of these studies, eight studies [42,55,56,59,60,63,64,65] had an annual cycle length while one study in the UK [58] used a 6-month cycle length. Three studies used individual sampling models (microsimulation models) which incorporated the patients’ unique medical histories and characteristics and complexities of the health system and multimorbidity in the modelling [43,57,66]. The study conducted in Uganda used an epidemiologic-cost model to estimate costs and effects of integrated screening and treatment [41]. One study conducted in the USA used a discrete event simulation (DES) for their evaluation from the health system and payer perspective [62]. One study conducted in the USA used a semi-Markov model in the evaluation to incorporate time-varying mortality [60].

##### Model perspective

Seven of the included studies performed the economic evaluations from a health care system perspective [41,42,56,57,61,63,66,67] while only three studies included a societal or patient perspective in their evaluations [55,58,62]. Three studies performed their evaluations from both health system and payer perspectives [43,58,62]. One study used a health care funder perspective [65] and another study in Germany was performed from the perspective of the German statutory health insurance [64].

#### Model horizon

The time horizons in the included studies ranged between two years and a lifetime horizon. Ten of the selected studies used a lifetime horizon [55,58,59,61,62,63,64,65,66,67]. Four studies conducted in the USA, Uganda, and Bhutan used a 10-year horizon [41,43,56,60], while one study in Kenya used a 15-year horizon [57]. In addition to a lifetime horizon, three studies in Taiwan, UK, and USA simulated between one and 20 years horizon [43,58,67].

#### Model adaptations

Eight of the selected studies either adapted existing models or used them to generate inputs. One study conducted in Australia [42] used the UK Prospective Diabetes Study (UKPDS) outcomes model to estimate costs and effects of the alternative models of care [69]. Studies conducted in Bhutan (Mousa) and the Netherlands [61] used the UKPDS risk engine to calculate CVD risk estimates for their models [70,71]. A study conducted in the USA [43] and another in six European countries and the UK [66], used the Archimedes model to estimate costs and quality-adjusted life years (QALYs). The Archimedes model is validated and has been widely used to model diabetes [72,73]. The study in Uganda [41] used the Globorisk model to obtain estimates of CVD risk, while one study in the USA [60] used Framingham risk equations [74]. The study conducted in Kenya used an existing population-based model of HIV dynamics (SPECTRUM) combined with a microsimulation [57].

### Quality assessment

The mean quality score in the included studies was 81% [57%–93%] (Supplementary table 7). The selected studies showed good quality of reporting on the model structure domain (89%). The studies showed adequate reporting and justification of the decision problem and objective, perspective and scope of the model, rationale for the structure, assumptions, model type, horizon, cycle length and disease pathways. However, only four studies provided a clear statement on whether all feasible and practical options were evaluated [41,55,64,67] while it was not clear in 11 studies. Despite almost 90% of studies stating the model perspective, the primary decision-maker for the analysis was not clear in almost half of the selected studies. Majority of studies adequately reported costs, utilities and their sources, data identification, and methods of incorporating data (76%). Notably, patient indirect costs and productivity losses were not reported for most studies, including those that used a societal perspective. Furthermore, only one study evaluating a quality improvement collaborative reported program implementation costs such as project management and local overhead costs for participating facilities [61]. However, deficiencies were noted in the assessment of uncertainty where only two studies reported having used all the four principal types of uncertainty (methodological, structural, parameter, and heterogeneity) with the majority of studies not justifying omission [58,62]. Only five out of the 16 selected studies performed subgroup analysis by disaggregating the final cost-effectiveness results by gender or age group [41,57,58,62,64]. More than 80% of the studies adequately reported and addressed structural and parameter uncertainty while less than half assessed methodological uncertainty or heterogeneity by running the models separately for different subgroups. For the consistency domain, higher scores were achieved for external consistency as compared to internal consistency. Only five studies justified the internal validity of their model [42,43,61,62,66].

## Discussion

A total of 16 studies were included in this systematic review, the majority of which were conducted in HICs rather than LMICs. There was significant heterogeneity in the types of integrated care models evaluated as well as in the chronic disease focus of the included studies. However, most of the studies used cohort Markov models and focused on hypertension or diabetes as the primary disease condition. While the health outcome metrics reported were consistent, there was poor reporting of data validation approaches against local data, the quality of data incorporated in the models, and internal and external consistency. While only three of the selected studies used individual patient simulations, they were better able to capture complexities of integrated care interventions and multimorbidity.

This systematic review adopted a narrative synthesis without a quantitative meta-analysis to report the results from the included studies. The decision not to undertake a meta-analysis for this study was driven by the substantial heterogeneity across the included studies that rendered statistical pooling inappropriate. Specifically, the studies varied markedly in the types and combinations of integrated care interventions evaluated (e.g., integrated screening, pharmacist-led care, nurse-led clinics, quality improvement collaboratives), the underlying decision-analytic model structures evaluated (including cohort-based Markov models, microsimulation models, discrete event simulation, and epidemiologic–cost models), and the disease areas and multimorbidity patterns modelled in the specific studies. In addition, outcome measures differed across studies, with some reporting cost per QALY gained, others reporting cost per life-year gained or disease-specific outcomes, and variation in time horizons, perspectives, discount rates, and comparators. Given this methodological and conceptual diversity, a meta-analysis would risk producing misleading summary estimates and obscure important contextual and structural differences between models. Consequently, a narrative synthesis was deemed the most appropriate approach to critically appraise how decision-analytic models have been applied, rather than to quantitatively pool cost-effectiveness results that are not directly comparable.

The majority of the integrated care models in the included studies were integrated screening and treatment interventions at health facility and community level. This is consistent with existing literature on integrated care for multiple chronic diseases focused on integrating screening and treatment [75,76,77,78]. The primary disease conditions in the evaluations were diabetes or hypertension which supports literature from existing studies on integrated care [79,80,81,82]. The only two studies from this review focused on infectious disease and NCD integration were conducted in SSA countries. This corroborates studies on integrated care in SSA which have focused on HIV and NCD integration in the region [82,83,84,85]. A plausible reason for this integration is the heightened risk of NCDs among people living with Human immunodeficiency virus (PLHIV) who have higher life expectancy due to improved therapies and management structures. However, the findings indicate the need to evaluate models of integration for concordant cardiometabolic multimorbidity occasioned by the rising burden of these conditions [21].

The majority of the studies performed the model-based economic evaluations from a health system perspective [41,42,56,57,61,63,66,67], while only three studies included a societal or patient perspective in their evaluation [55,58,62]. Of note is that the studies that included a patient perspective did not report including the indirect costs such as informal care and productivity losses in addition to the direct patient costs as recommended by existing health economic guidelines [86]. Most of the selected studies did not provide implementation costs of the integrated care models. In fact, only one study that evaluated a quality improvement collaborative included the program implementation costs, such as program management costs and local overhead costs for each participating facility, as part of their inputs for the economic model [61]. Guidelines for economic evaluations recommend a lifetime horizon for interventions that impact costs and outcomes over a patient’s lifetime, with shorter horizons being appropriately justified [86]. However, a third of the selected studies did not include a lifetime horizon and studies that used 10-year [41,43,56,60] and 15-year [57] horizons did not provide clear justifications. Due to the long-term effects of multiple chronic conditions and hence the need to seek continuous care, a lifetime horizon is appropriate in economic evaluations of integrated care.

Most of the DAMs in the selected studies were Markov models. The evaluation of a chronic disease program in Korea used a Markov model that assumed identical progression probabilities after development of complications in the two groups, which may underestimate or overestimate the outcomes [59]. Similarly, a Markov model to evaluate the WHO PEN model for hypertension and diabetes in Bhutan assumed similar treatment outcomes for comorbidity and diabetes alone [55], yet there may be considerable variation in the disease outcomes for a single disease compared to a patient with comorbidity. Cohort Markov models are considered limited in assessing complex health care interventions due to their limitations in capturing individual dynamics [39,87,88]. In the case of integrated care for multiple chronic diseases, the multiple disease states that would exist with multimorbidity and complex interactions within an integrated health system, such as repeated interactions with health care, may not be adequately represented. Furthermore, assumptions of constant transition probabilities, as was observed with majority of the selected studies using Markov models, overlook potential temporal changes and relationships that may exist, and important interplays between health system elements and patient behaviours may be a potential limitation. For such interventions, more advanced methodologies such as individual sampling models, dynamic simulation, or system dynamics models are recommended to better encompass the complexities [39,89]. For instance, one of the included studies conducted in Kenya [57] used a microsimulation model and was able to model CVD dynamics at individual level by modelling impact on patients based on their levels of CVD risk, which was not captured by the cohort level models.

Despite most of the included DAMs scoring well in their quality of reporting, there was poor reporting with regards to accounting for uncertainty and validation in the studies. Philips et al., (2006) recommend distinguishing between the four principal types of uncertainty when reporting economic evaluations i.e. methodological, structural, heterogeneity, and parameter uncertainty [54]. Similar to our findings, other reviews on economic evaluations show that economic evaluations have scored highly on reporting structural and parameter uncertainty through one-way, two-way, and probabilistic sensitivity analyses, and scoring poorly on reporting heterogeneity and methodological uncertainty [90,91]. With regards to heterogeneity, only about a third of the selected articles reported performing subgroup analysis. For a health care intervention such as integrated care which impacts the general population, it is important to assess its differential impact on different groups to account for the equity considerations in case the interventions are to be scaled up, an important consideration for policy makers.

The findings of our systematic review have important implications for health care decision making for chronic disease prevention and control in LMICs where the burden of multimorbidity is increasing [21,92]. Given the demonstrated potential benefits of integrated care for patients with chronic disease multimorbidity in other settings, the scarcity of economic evaluations using DAMs in LMICs signals a substantial data gap that limits evidence-based decision making for chronic disease prevention and control. Therefore, there is an urgent need for more economic evaluations using DAMs to inform integrated care models of healthcare delivery that are tailored to different contexts in LMICs for cardiometabolic multimorbidity and other common multimorbidity patterns. Considering the social and health inequalities and disparities in healthcare provision that have been evidenced in LMICs [93,94], health economists and analysts should consider the use of individual sampling models that will capture the complexity of different integrated care configurations and patients interactions with the health care models in the context of multiple diseases using a lifetime horizon. This ensures that recommended integrated care models are deemed cost-effective, context-specific, and sustainable.

This systematic review is the first to synthesise evidence on model-based economic evaluations conducted to evaluate integrated care for cardiometabolic multimorbidity. The key strength of our study is the broad review of the literature that was designed to capture studies with a wide heterogeneity and synthesized the findings to provide more understanding of how integrated care can been modelled using DAMs. Our study has some limitations. Firstly, we only included original articles published in the English language, hence there is a chance that relevant studies published in other languages may have inadvertently been excluded from the review. Furthermore, non-inclusion of grey literature such as PhD or master’s student theses, reports or guidance documents may have left out some relevant studies that meet the inclusion criteria. Despite these limitations, our systematic review contributes to the existing literature by synthesizing and appraising the current evidence base on the use of DAMs to model the economic impact of integrated care interventions for cardiometabolic multimorbidity.

## Conclusion

Model-based economic evaluations (including simulated Markov models, individual sampling models, discrete event simulation, and epidemiologic cost models) have been used to evaluate integrated care interventions for cardiometabolic multimorbidity, most often considering hypertension and/or diabetes in high-income countries. Future studies could improve in their consideration of uncertainty and validation and should consider methods such as microsimulation that can more easily describe repeated interactions with health care. More studies should consider inclusion of patient costs, which are a domain of potential benefit from integrated care. More studies using DAMs to evaluate integrated care for cardiometabolic multimorbidity are needed to inform decision making in LMICs, especially individual sampling models that capture the complexity of integrated care delivery within specific contexts for different populations with multimorbidity.

## Additional File

The additional file for this article can be found as follows:

10.5334/ijic.9075.s1 Supplementary Material. File contains details on Search strategy, definition of terms, inclusion and exclusion criteria, detailed description of study selection, characteristics of included studies, integrated care components in the included studies, summary of economic evaluation results, and results of quality assessment of included studies.

## Reviewers

Cam Donaldson PhD FRSE, Emeritus Yunus Chair, Yunus Centre for Social Business & Health, Glasgow Caledonian University, UK.

Dr. med. Hendrik Napierala MSc, Charité – Universitätsmedizin Berlin, corporate member of Freie Universität Berlin and Humboldt-Universität zu Berlin, Institute of General Practice, Berlin, Germany.

Maedeh Sharafoddin, Orthopedic Research Center, Mashhad University of Medical Sciences, Mashhad, Iran and Bone and Joint Research Laboratory, Ghaem Hospital, Mashhad University of Medical Sciences, Mashhad, Iran.
