# Supplementary material for: Application of Decision-Analytic Models to Evaluate Integrated Care Interventions for Cardiometabolic Multimorbidity: A Systematic Review
Source: Int J Integr Care. 2026 Apr 27;26(2):7. doi: 10.5334/ijic.9075 (PMC13131342; doi:10.5334/ijic.9075)
Supplement: Supplementary Material. — File contains details on Search strategy, definition of terms, inclusion and exclusion criteria, detailed description of study selection, characteristics of included studies, integrated care components in the included studies, summary of economic evaluation results, and results of quality assessment of included studies. [file ijic-26-2-9075-s1.pdf]

Supplementary material for "Decision-analytic models to inform integrated care interventions for cardiometabolic multimorbidity: A systematic review" by Wambiya *et al.*

Supplementary note 1: Search strategies for electronic databases

1. **Medline search Strategy**

1 ((effectiveness or cost effective* or microsim* or simulation or cost utility or cost-utility or cost minimi#ation or cost-minimi#ation or Markov or agent-based or mathematical or cost benefit or cost-benefit) adj model*).tw.

2 exp Models, Mathematical/

3 (decision adj1 (tree$ or analy$ or model$)).tw

4 exp "Costs and Cost Analysis"/

5 exp Cost-Benefit Analysis/

6 (economic adj (evaluation* or impact)).tw.

7 or/1-6

8 exp "Delivery of Health Care, Integrated"/

9 exp Comprehensive Health Care/

10 exp "Continuity of Patient Care"/

11 exp Patient-Centered Care/

12 exp "Referral and Consultation"/

13 (referral adj consultation).tw

14 ((transmural or seamless) adj care).tw.

15 exp Patient Care Team/

16 ((integrat* or coordinat* or horizontal or vertical) adj2 (care or service* or program* or deliver* or management)).tw.

17 (multi team or multiteam or multi care or multicare or multi clinic or multiclinic or multi service or multiservice or multi program* or multiprogram*or multi delivery or multidelivery or multi management).tw.

18 or/8-17

19 exp Chronic Disease/

20 (chronic adj2 (condition* or illness* or disease* or disorder*)).tw.

21 exp Noncommunicable Diseases/

22 non-communicable disease.mp

23 or/19-22

24 exp Metabolic Syndrome/ or exp Hypertension/ or exp Cardiovascular Diseases/ or exp Obesity/ or exp Metabolic Diseases/ or exp Diabetes Mellitus, Type 2/

25 kidney disease/

26 (Cardiovascular or cardio-vascular or CVD or heart disease* or cardiometabolic or cardio metabolic or stroke or cerebrovasc* or circulatory disease or myocardial infarction or arteriosclero* or atherosclero* or CIMT or (carotid adj2 (intima-media or intima media or IMT or plaque)) or blood pressure or hypertens* or pulse wave velocity or augmentation index or arterial stiffness or arterial stiffening or metabolic syndrome or diabetes or fasting glucose or insulin or cholesterol or lipid profile or lipid* or triglyceride* or obesity).tw.

27 or/24-26

28 and/7,18,23,27 not regression.tw. not qualitative.tw. not survey.tw.

29 limit 28 to english language (**957 articles**)

2. **Embase search Strategy 1974 to 2023 Week 49**

1 ((effectiveness or cost-effective* or microsim* or simulation or cost utility or cost-utility or cost minimi#ation or cost-minimi#ation or Markov or agent-based or mathematical or cost benefit or cost-benefit) adj model*).tw. 115243

2 exp Mathematical model/ 915951

3 exp "Costs and Cost Analysis"/ 410272

4 exp Cost-Benefit Analysis/ 95511

5 (economic adj (evaluation* or impact)).tw. 39528

6 (decision adj1 (tree$ or analy$ or model$)).tw. 38406

7 1 or 2 or 3 or 4 or 5 or 6 1478842

8 exp "Delivery of Health Care, Integrated"/ 13747

9 exp Comprehensive Health Care/ 6545478

10 exp "Continuity of Patient Care"/ 1039565

11 exp Patient-Centered Care/ 1039565

12 exp "Referral and Consultation"/ 160588

13 (referral adj consultation).tw. 88

14 ((transmural or seamless) adj care).tw. 462

15 exp Patient Care Team/ 7175

16 ((integrat* or coordinat* or horizontal or vertical) adj (care or service* or program* or deliver* or management)).tw. 24611

17 (multi team or multiteam or multi care or multicare or multi clinic or multiclinic or multi service or multiservice or multi program* or multiprogram*or multi delivery or multidelivery or multi management).tw. 882

18 8 or 9 or 10 or 11 or 12 or 13 or 14 or 15 or 16 or 176553871

19 exp Chronic Disease/ 245087

20 (chronic adj2 (condition* or illness* or disease* or disorder*)).tw. 502381

21 exp Noncommunicable Diseases/ 12057

22 non-communicable disease.mp. [mp=title, abstract, heading word, drug trade name, original title, device manufacturer, drug manufacturer, device trade name, keyword heading word, floating subheading word, candidate term word] 14157

23 19 or 20 or 21 or 22 670845

24 exp Metabolic Syndrome/ or exp Hypertension/ or exp Cardiovascular Diseases/ or exp Obesity/ or exp Metabolic Diseases/ or exp Diabetes Mellitus, Type 2/ 7503141

25 kidney disease/ 131142

26 (Cardiovascular or cardio-vascular or CVD or heart disease* or cardiometabolic or cardio metabolic or stroke or cerebrovasc* or circulatory disease or myocardial infarction or arteriosclero* or atherosclero* or CIMT or (carotid adj2 (intima-media or intima media or IMT or plaque)) or blood pressure or hypertens* or pulse wave velocity or augmentation index or arterial stiffness or arterial stiffening or metabolic syndrome or diabetes or fasting glucose or insulin or cholesterol or lipid profile or lipid* or triglyceride* or obesity).tw. 4363534

27 24 or 25 or 26 8792333

28 7 and 18 and 23 and 27 11882

29 limit 28 to (english language and embase and article) 4546

30 (7 and 18 and 23 and 27) not regression.tw. not qualitative.tw. not survey.tw. 5790

31 limit 30 to (english language and embase and article) **2637 articles**

3. Web of Science Search strategy

1 effectiveness model* or cost effective* model* or microsim* model* or simulation model* or cost utility model* or cost-utility model* or cost minimi$ation model* or cost-minimi$ation model* or Markov model* or agent-based model* or mathematical model* or cost benefit model* or cost-benefit model*

2 Cost Analysis

3 Cost-Benefit Analysis

4 economic NEAR (evaluation* or impact)

5 (decision NEAR (tree$ or analy$ or model$))

6 #5 OR #4 OR #3 OR #2 OR #1

7 Delivery of Health Care

8 Comprehensive Health Care

9 Continuity of Patient Care

10 Patient-Centered Care

11 referral NEAR consultation

12 (transmural or seamless) NEAR care

13 Patient Care Team

14 ((integrat* or coordinat* or horizontal or vertical) NEAR (care or service* or program* or deliver* or management))

15 (multi team or multiteam or multi care or multicare or multi clinic or multiclinic or multi service or multiservice or multi program* or multiprogram*or multi delivery or multidelivery or multi management)

16 #7 OR #8 OR #9 OR #10 OR #11 OR #12 OR #13 OR #14 OR #15

17 Chronic Disease

18 (chronic NEAR (condition* or illness* or disease* or disorder*))

19 non-communicable disease

20 #17 or #18 or #19

21 Metabolic Syndrome or Hypertension or Cardiovascular Diseases or Obesity or Metabolic Diseases or Diabetes*

22 kidney disease

23 (Cardiovascular or cardio-vascular or CVD or heart disease* or cardiometabolic or cardio metabolic or stroke or cerebrovasc* or circulatory disease or myocardial infarction or arteriosclero* or atherosclero* or CIMT or (carotid adj2 (intima-media or intima media or IMT or plaque)) or blood pressure or hypertens* or pulse wave velocity or augmentation index or arterial stiffness or arterial stiffening or metabolic syndrome or diabetes or fasting glucose or insulin or cholesterol or lipid profile or lipid* or triglyceride* or obesity)

24 #21 or #22 or #23

25 #6 AND #16 AND #20 AND #24 NOT regression or qualitative or survey

26 limit 25 to english language and article and Web of science core collection (**2746 articles**)

4. Cochrane Library search strategy

1 ((effectiveness or cost effective* or microsim* or simulation or cost utility or cost-utility or cost minimi#ation or cost-minimi#ation or Markov or agent-based or mathematical or cost benefit or cost-benefit) NEAR model*)

2 (decision NEAR (tree# or analy* or model*))

3 Costs and Cost Analysis

4 Cost-Benefit Analysis

5 economic evaluation

6 economic impact

7 #1 OR #2 OR #3 OR #4 OR #5 OR #6

8 “Delivery of Health Care”

9 “Comprehensive Health Care”

10 “Continuity of Patient Care”

11 “Patient Centered Care”

12 "Referral and Consultation"

13 (referral adj consultation)

14 “transmural care”

15 “seamless care”

16 “Patient Care Team”

17 (integrat* or coordinat* or horizontal or vertical) NEAR (care or service* or program* or deliver* or management)

18 "multi team" or "multiteam" or "multi care" or "multicare" or "multi clinic" or "multiclinic " or "multi service" or "multiservice" or multi program* or multiprogram* or "multi delivery" or "multidelivery" or "multi management"

19 #8 OR #9 OR #10 OR #11 OR #12 OR #13 OR #14 OR #15 OR #16 OR #17 OR #18

20 Chronic Disease

21 (chronic NEAR (condition* or illness* or disease* or disorder*))

22 non-communicable disease

23 #20 or #21 or #22

24 Metabolic Syndrome or Hypertension or Cardiovascular Diseases or Obesity or Metabolic Diseases or Diabetes*

25 kidney disease

26 (Cardiovascular or cardio-vascular or CVD or heart disease* or cardiometabolic or cardio metabolic or stroke or cerebrovasc* or circulatory disease or myocardial infarction or arteriosclero* or atherosclero* or CIMT or (carotid adj2 (intima-media or intima media or IMT or plaque)) or blood pressure or hypertens* or pulse wave velocity or augmentation index or arterial stiffness or arterial stiffening or metabolic syndrome or diabetes or fasting glucose or insulin or cholesterol or lipid profile or lipid* or triglyceride* or obesity)

27 #24 or #25 or #26

28 #7 AND #19 AND #23 AND #27 NOT regression or qualitative or survey (**241 articles**)

5. CINAHL search strategy

1 (effectiveness or cost effective* or microsim* or simulation or cost utility or cost-utility or cost minimi#ation or cost-minimi#ation or Markov or agent-based or mathematical or cost benefit or cost-benefit)

2 Mathematical model

3 Costs and Cost Analysis

4 Cost Benefit Analysis

5 economic evaluation

6 economic impact

7 (decision NEAR (tree# or analy* or model*))

8 #1 OR #2 OR #3 OR #4 OR #5 OR #6 OR #7

9 “Delivery of Health Care”

10 “Comprehensive Health Care”

11 “Continuity of Patient Care”

12 “Patient Centered Care”

13 "Referral and Consultation"

14 referral N2 consultation

15 “transmural care”

16 “seamless care”

17 “Patient Care Team”

18 (integrat* or coordinat* or horizontal or vertical) N2 (care or service* or program* or deliver* or management)

19 "multi team" or "multiteam" or "multi care" or "multicare" or "multi clinic" or "multiclinic " or "multi service" or "multiservice" or multi program* or multiprogram* or "multi delivery" or "multidelivery" or "multi management"

20 #9 OR #10 OR #11 OR #12 OR #13 OR #14 OR #15 OR #16 OR #17 OR #18 OR #19

21 Chronic Disease

22 (chronic NEAR (condition* or illness* or disease* or disorder*))

23 non-communicable disease

24 #21 OR #22 OR #23

25 Metabolic Syndrome or Hypertension or Cardiovascular Diseases or Obesity or Metabolic Diseases or Diabetes*

26 kidney disease

27 (Cardiovascular or cardio-vascular or CVD or heart disease* or cardiometabolic or cardio metabolic or stroke or cerebrovasc* or circulatory disease or myocardial infarction or arteriosclero* or atherosclero* or CIMT or (carotid adj2 (intima-media or intima media or IMT or plaque)) or blood pressure or hypertens* or pulse wave velocity or augmentation index or arterial stiffness or arterial stiffening or metabolic syndrome or diabetes or fasting glucose or insulin or cholesterol or lipid profile or lipid* or triglyceride* or obesity)

28 #25 OR #26 OR #27

29 #8 AND #20 AND #24 AND #28 NOT regression or qualitative or survey

30 Limit to English (**267 articles**)

6. **APA PsychInfo via Ovid search strategy**

1 ((effectiveness or cost effective* or microsim* or simulation or cost utility or cost-utility or cost minimi#ation or cost-minimi#ation or Markov or agent-based or mathematical or cost benefit or cost-benefit) adj model*).tw.

2 exp Models/

3 (decision adj1 (tree$ or analy$ or model$)).tw

4 exp "Costs and Cost Analysis"/

5 Cost-Benefit Analysis.tw

6 (economic adj (evaluation* or impact)).tw.

7 or/1-6

8 exp "Delivery of Health Care, Integrated"/

9 exp Comprehensive Health Care/

10 exp "Continuity of Patient Care"/

11 exp Patient-Centered Care/

12 exp "Referral and Consultation"/

13 (referral adj consultation).tw

14 ((transmural or seamless) adj care).tw.

15 exp Patient Care Team/

16 ((integrat* or coordinat* or horizontal or vertical) adj2 (care or service* or program* or deliver* or management)).tw.

17 (multi team or multiteam or multi care or multicare or multi clinic or multiclinic or multi service or multiservice or multi program* or multiprogram*or multi delivery or multidelivery or multi management).tw.

18 or/8-17

19 exp Chronic Disease/

20 (chronic adj2 (condition* or illness* or disease* or disorder*)).tw.

21 exp Noncommunicable Diseases/

22 non-communicable disease.mp

23 or/19-22

24 exp Metabolic Syndrome/ or exp Hypertension/ or exp Cardiovascular Diseases/ or exp Obesity/ or exp Metabolic Diseases/ or exp Diabetes Mellitus, Type 2/

25 kidney disease/

26 (Cardiovascular or cardio-vascular or CVD or heart disease* or cardiometabolic or cardio metabolic or stroke or cerebrovasc* or circulatory disease or myocardial infarction or arteriosclero* or atherosclero* or CIMT or (carotid adj2 (intima-media or intima media or IMT or plaque)) or blood pressure or hypertens* or pulse wave velocity or augmentation index or arterial stiffness or arterial stiffening or metabolic syndrome or diabetes or fasting glucose or insulin or cholesterol or lipid profile or lipid* or triglyceride* or obesity).tw.

27 or/24-26

28 and/7,18,23,27 not regression.tw. not qualitative.tw. not survey.tw.

29 limit 28 to english language (**13 articles**)

Scopus search strategy

(TITLE-ABS-KEY

(effectiveness OR cost AND effective* OR microsim* OR simulation OR cost AND utility OR cost-utility OR cost AND minimi?ation OR cost-minimi?ation OR markov OR agent-based OR mathematical OR cost AND benefit OR cost-benefit) OR TITLE-ABS-KEY (mathematical W/2 model) OR TITLE-ABS-KEY (costs AND cost AND analysis) OR TITLE-ABS-KEY (cost AND benefit AND analysis) OR TITLE-ABS-KEY (economic AND evaluation) OR TITLE-ABS-KEY (economic AND impact) OR TITLE-ABS-KEY ((decision W/2 (tree# OR analy* OR model*)))) AND (TITLE-ABS-KEY ("Delivery of Health Care") OR TITLE-ABS-KEY ("Comprehensive Health Care") OR TITLE-ABS-KEY ("Continuity of Patient Care") OR TITLE-ABS-KEY ("Patient Centered Care") OR TITLE-ABS-KEY ("Referral and Consultation") OR TITLE-ABS-KEY (referral W/2 consultation) OR TITLE-ABS-KEY ("transmural care") OR TITLE-ABS-KEY ("seamless care") OR TITLE-ABS-KEY ("Patient Care Team") OR TITLE-ABS-KEY ((integrat* OR coordinat* OR horizontal OR vertical) PRE/2 (care OR service* OR program* OR deliver* OR management)) OR TITLE-ABS-KEY ("multi team" OR "multiteam" OR "multi care" OR "multicare" OR "multi clinic" OR "multiclinic " OR "multi service" OR "multiservice" OR multi AND program* OR multiprogram* OR "multi delivery" OR "multidelivery" OR "multi management")) AND (TITLE-ABS-KEY

(chronic AND disease) OR TITLE-ABS-KEY ((chronic PRE/3 (condition* OR illness* OR disease* OR disorder*))) OR TITLE-ABS-KEY (non-communicable AND disease)) AND (TITLE-ABS-KEY (metabolic AND syndrome OR hypertension OR cardiovascular AND diseases OR obesity OR metabolic AND diseases OR diabetes*) OR TITLE-ABS-KEY (kidney AND disease) OR TITLE-ABS-KEY ((cardiovascular OR cardio-vascular OR cvd OR heart AND disease* OR cardiometabolic OR cardio AND metabolic OR stroke OR cerebrovasc* OR circulatory AND disease OR myocardial AND infarction OR arteriosclero* OR atherosclero* OR cimt OR blood AND pressure OR hypertens* OR pulse AND wave AND velocity OR augmentation AND index OR arterial AND stiffness OR arterial AND stiffening OR metabolic AND syndrome OR diabetes OR fasting AND glucose OR insulin OR cholesterol OR lipid AND profile OR lipid* OR triglyceride* OR obesity)) OR TITLE-ABS-KEY ((carotid PRE/2 intima-media) OR (carotid PRE/2 intima AND media) OR (carotid PRE/2 imt) OR (carotid PRE/2 plaque))) AND (LIMIT-TO (DOCTYPE , "ar")) AND (LIMIT-TO (LANGUAGE , "English"))

(**338 articles**)

Supplementary table 1: Definition of terms

Terms	Definition
**Cost-effectiveness analysis**	A form of comparative economic analysis that evaluated two or more alternatives in terms of relative cost and outcomes, where the outcomes are measured in a single natural unit (e.g., life years gained, disease case averted, new cases detected)
**Cost-utility analysis**	A type of cost-effectiveness analysis where the outcomes are expressed by a generic measure of health status that considers both the effect on mortality and morbidity e.g. quality-adjusted life years (QALYs) and disability-adjusted life years (DALYs))
**Cardiometabolic multimorbidity**	The existence of two or more chronic diseases in the same individual, at least one of which was a cardiometabolic disease including cardiovascular diseases (e.g., coronary heart disease, cerebrovascular disease, peripheral arterial disease, rheumatic heart disease, congenital heart disease, and deep vein thrombosis), metabolic syndrome, diabetes mellitus, and hypertension in the same individual.
**Concordant multimorbidity**	The co-existence of two or more chronic diseases all of which

	are cardiometabolic diseases. i.e., are pathophysiologically related and have similar management approaches and treatment plans (e.g., type 2 diabetes and hypertension)
**Discordant multimorbidity**	The existence of two or more chronic diseases at least one of which is a cardiometabolic disease i.e., pathophysiologically unrelated and require different management approaches (for example type 2 diabetes and HIV).
**Integrated care**	Health service delivery containing two or more components of the chronic care model (CCM), as defined by Wagner [64,65], and in line with previous research for the management of cardiometabolic multimorbidity, and at least one element of Singer et al., (2011) [66] framework for measuring integrated patient care for patients with multiple or complex chronic conditions.
**Decision analytic model**	A mathematical framework that uses quantitative relationships to define a series of possible consequences arising from a set of alternative options being evaluated, in order to synthesise evidence on costs and health outcomes. [46-49]
**Health care system/ provider perspective**	A viewpoint for an economic evaluation that includes only direct medical costs incurred by the healthcare provider or system, such as costs for hospitals, staff, drugs, and consultations. [95]

**Societal perspective**	A comprehensive viewpoint for an economic evaluation that includes all costs and benefits, regardless of who incurs or receives them. This includes direct medical costs, direct non-medical costs (e.g., patient travel, informal care), and indirect costs such as productivity losses due to morbidity or mortality. [95]
**Payer perspective**	A viewpoint for an economic evaluation that includes all costs covered by a third-party payer, such as an insurance company or a national health service, typically including direct medical costs. [95]
**Cohort Markov Model/ Simulated Markov model**	An aggregate-level model that simulates the progression of a hypothetical cohort of patients through a finite set of mutually exclusive health states over discrete time periods (cycles). The model is "simulated" because Monte Carlo methods are used to estimate outcomes by sampling transitions for the cohort, which can account for parameter uncertainty. [48, 96]
**Simulated Patient Level Markov Model**	An individual-level model that simulates the progression of single patients through a set of health states over time. It can account for patient heterogeneity by assigning unique attributes to each individual but retains the Markovian assumption that the future depends only on the current state. [48]
**Epidemiological-cost model**	A type of decision model that combines epidemiological data on disease incidence, prevalence, and risk with cost data to

	estimate the health and economic impacts of an intervention, often using simpler calculations rather than simulating individual patient trajectories over time. [50]
**Individual sampling model/ Microsimulation model**	An individual-level model that simulates the experiences of many distinct individuals one by one, allowing for a high degree of patient heterogeneity and complex patient histories. These models can be Markovian or non-Markovian and may or may not allow for interactions between individuals. [48]
**Discrete event simulation (DES)**	A highly flexible, individual-level modelling technique that represents entities (e.g., patients) as they progress through a system and compete for constrained resources (e.g., doctors, beds). Events occur at discrete points in time and can be sampled from any distribution, allowing for complex interactions and non-Markovian processes. [48]

Note: Numbering of the references cited in this table relates to those listed in the main manuscript.

Supplementary table 2: Inclusion and exclusion criteria according to the PICO framework

	Inclusion	Exclusion
**Population**	• Adults (18 years and above) at risk of or having either concordant or discordant cardiometabolic multimorbidity	• Individuals below 18 years, adolescents, and children.
**Intervention**	• Integrated care models or interventions in health care delivery as per the study definitions	• Interventions not considered integrated care as per the study definition
**Comparator**	• Alternative integrated care models or interventions<br>• Usual care for patients with chronic diseases	
**Outcomes**	• Cardiometabolic	• Single-disease focused studies

	multimorbidity as per the study definition	and other types of multimorbidity that do not include at least one cardiometabolic disease.

Supplementary note 2: Detailed description of the study selection process

The study selection process commenced with the import of searched outputs into the Endnote citation manager, where duplicates were meticulously handled. Initial elimination prioritized duplicates based on titles, followed by a more thorough examination involving author names and publication year to ensure comprehensive duplication identification.

Post-duplicate removal, the articles were exported as an XML file into Covidence software, chosen for its user-friendly interface. Covidence facilitated efficient screening by streamlining the eligibility assessment process, promoting collaboration among reviewers, and ensuring transparency in study selection.

The next phase involved independent screening by four reviewers (EW, JO, CA), each utilizing a predefined selection checklist. Titles and abstracts were rigorously assessed, with those meeting the eligibility criteria proceeding to full-text screening. Throughout this process, reviewers remained blinded to each other's decisions, enhancing the objectivity of the study selection. Any conflicts identified during screening were resolved through thorough discussion with a third reviewer (either of PD, RA, DG and PO), ensuring consistency and reliability.

The study selection adhered to predefined inclusion and exclusion criteria outlined in the systematic review protocol, contributing to the overall robustness of the systematic review. The PRISMA flow diagram in the main document visually represents the number of records identified, screened, assessed for eligibility, and ultimately included in the systematic review.

This comprehensive procedure, characterized by transparency and thoroughness, ensures the reliability and reproducibility of the study selection process.

Supplementary table 3: Characteristics of the included studies

Author (year)	Country	Evaluation type	Health system context	Integrated care intervention	Comparator	Primary disease modelled
Afzali et al. (2012) [77]	Australia	CUA	Primary health care	High level Practice nurse involvement in clinic-based activities	Low level Practice nurse involvement in clinic-based activities	Diabetes
Dukpa et al. (2014) [64]	Bhutan	CEA	Primary health care	WHO PEN which uses an integrated approach to assess and manage cardiovascular risk using hypertension and diabetes as entry points	No screening	Hypertension and/or diabetes
Hirsch et al. (2017) [52]	USA	CEA	Primary health care	A collaborative endocrinologist-pharmacist Diabetes Intense Medical Management (DIMM) "Tune-Up" clinic for complex diabetes patients	Usual primary care physician (PCP) care	Diabetes
Hobbs et al. (2005) [71]	United Kingdom	CEA	Primary health care	Different screening strategies (targeted, whole-population screening and opportunistic screening with prompts)	No screening	Atrial fibrillation
Howard et al. (2010) [74]	Australia	CUA	Primary health care	Primary care based screening for CKD risk factors and improved management	Routine glucose and blood pressure control	CKD
Kasaie et al. (2020) [66]	Kenya	CEA	Primary health care	A joint community outreach campaign for screening and treatment of HIV, hypertension, and diabetes	Standard care	HIV
Kim et al. (2021) [68]	Republic of Korea	CUA	Primary health care	A chronic disease management program (CDMP) in patients with hypertension	Usual care	Hypertension

Mason et al. (2005) [72]	United Kingdom	CUA	Primary health care and hospital	Specialist nurse–led clinics provided to improve lipid and blood pressure control in diabetic patients	Usual care	Diabetes
Mousa et al. (2021) [65]	Jordan	CEA	Primary health care and hospital	Pharmacist-led care	Usual care	Diabetes
Penaloza-Ramos et al. (2016) [67]	United Kingdom	CUA	Primary health care	Self-monitoring and self-management of blood pressure in hypertensive patients	Usual care	Hypertension
Sando et al. (2020) [50]	Uganda	CEA	Primary health care	Integration of screening and treatment for hypertension, diabetes, and hypercholesterolemia into HIV care	Standard HIV care without NCD screening	HIV
Schaufler et al. (2010) [73]	Germany	CUA	Primary health care	Screening strategy for type 2 diabetes mellitus including early detection and secondary prevention	Routine clinical practice	Diabetes
Schouten et al. (2010) [70]	Netherlands	CUA	Primary health care	A quality improvement collaborative (QIC) focusing on patients with diabetes	Usual care	Diabetes
Schuetz et al. (2013) [75]	Denmark, France, Germany, Italy, Poland, and the United Kingdom	CUA	Primary health care	A vascular disease Health Checks program which integrates the prevention, early detection, and treatment of type 2 diabetes, hypertension, dyslipidemia, and smoking.	Usual care	Diabetes
Schultz et al. (2021) [69]	USA	CUA	Primary health care	A pharmacist-led medication therapy management (MTM) clinic	Usual care	Hypertension
Wang et al. (2006) [76]	Taiwan	CEA	Primary care and hospital	A community-based Integrated Screening (KCIS) program for	No screening	Cancers: colorectal, breast,

				chronic diseases		cervical and oral cancer

Notes: CEA - cost effectiveness analysis; CUA - cost utility analysis; CKD - chronic kidney disease; CVD - cardiovascular disease; MI - myocardial infarction; GI - gastrointestinal; CHD - coronary heart disease; CHF - congestive heart failure; IHD - Ischaemic heart disease; PEN - Package of essential NCD interventions

Supplementary table 4: Components of the integrated care models in the included studies

Author (year)	Community resources and policies	Health care organization	Self-management support	Delivery system design	Decision Support	Clinical information System	Coordinated care (between and within care teams)<br>Continuity of care<br>Patient-centred care
Afzali et al. (2012)		Organisation supports PN involvement in clinic-based activities	Provision of self-management services by practice nurses	Support for non-physician personnel (PN) involvement in self-management support and continuous monitoring of patients and the practice.			PN involvement in clinic-based activities (coordinated care)<br>Monitoring of progress (continuity of care)
Dukpa et al. (2014)		Organisations support the implementation of PEN interventions	Provision of education to promote self-management	Training of non-physician and primary care health care providers<br>Creating practice teams to implement the PEN	PEN guidelines focused on lifestyle modification		Coordination between non-physician and physicians involved in delivering the PEN (Coordinated care)

				model			
Hirsch et al. (2017)		Organisation support the pharmacist-led MTM	Provision of patient-specific diabetes education	Support for pharmacist and endocrinologist activities related to education, collaborative care plan, and interpreting laboratory tests	MTM Development of a personalised care plan using an MTM Spider Web model approach, which considers each patient's comorbidities, complications, and clinical, socioeconomic, and behavioural issues		Coordination between pharmacist, endocrinologist, and other PCPs (coordinated care) Personalised care plan developed using MTM spider web model approach that considers each patient's comorbidities, complications, and clinical, socioeconomic, and behavioural issues (patient-centred care)

							Provision of specific diabetes education during approximately three 60-minute visits over a 6-month period (Continuity of care)
Hobbs et al. (2005)		Organisation supports systematic screening of AF		GPs and practice nurses in the intervention practices received education on the importance of AF detection and ECG interpretation	Screening and education guidelines for the opportunistic screening program		Coordination between GPs and PNs for systematic screening of AF (Coordinated care)
Howard et al. (2010)		Organisations support Improved management of known patients with CKD risk		Support for providers to provide improved management and primary care-based screening for CKD risk factors			Intensive glucose and BP control (Patient-centred care)

		factor and Primary care–based screening strategies for CKD risk factors					
Kasaie et al. (2020)	Multi Disease health campaigns under large tents in all communities during weekdays, evenings, and weekends in collaboration with local health units and the	Program supports the community-based, multi-disease testing and treatment strategy		Support to healthcare providers to conduct multi disease health campaigns under large tents in all communities during weekdays, evenings, and weekends in collaboration with local health units and provide referral for patients found with HIV, hypertension, or diabetes	Use of the 2015 World Health Organization ART guidelines in which universal ART was recommended	In addition to residents' names, biometric identifiers based on each resident's digital fingerprint were used to identify residents during their participation in testing and care activities in the community.	Coordination between health facilities and mobile clinics (coordinated care) Linkage to care for patients found to have HIV, hypertension, or diabetes (Continuity of care)

	Ministry of Health in Uganda and in Kenya Home-based testing for those who did not attend community campaigns						
Kim et al. (2021)		Primary care clinics support the CDMP	Provision of health support services, such as professional health consultation and education	Support and incentives provided for health care providers participating in the CDMP			Patients receive continued care (continuity of care) Provision of health support services, such as professional health consultation and education (Patient-

							centred care)
Mason et al. (2005)		Clinics supports the specialist nurse-led intervention to treat and control hypertension and hyperlipidemia in patients with diabetes	At subsequent visits, lifestyle factors were reinforced and reviewed, and medications were titrated according to response to the treatment and according to protocol.	Nurses received additional training in the management of hypertension and dyslipidemia in patients with diabetes from the local clinicians (J.M.G. and J.P.N.) and pharmacists	The use of existing guidelines to treat and control hypertension and hyperlipidemia in patients with diabetes		Coordination between nurses clinicians, and pharmacists (coordinated care) Individualised action based on the hospital visit (Person-centred care)

Mousa et al. (2021)		Organisations support pharmacist-led care	Pharmacists provided medication counselling, offered instructions on self-monitoring, and advised patients on healthy lifestyle choices	Support for pharmacist-led care for patients with type 3 diabetes	Guidelines for medication prescription and procedures in patients with T2D		Coordination between pharmacists and other health care providers (Coordinated care) Monitoring drug adherence (Continuity of care)
Penaloza-Ramos et al. (2016)		Health facility support the self-management program	Patients randomly assigned to self-management were trained to self-monitor BP and to self-titrate their antihypertensive medication	Support to enable patient to communicate with family physician from home			Patients attended two or three sessions, each lasting around an hour and monitored monthly (Continuity of care)

Sando et al. (2020)		HIV clinics support the NCD screening program		Support to healthcare providers in HIV clinics to conduct NCD screening	The use of NCD screening guidelines		Management of patients with NCDs at the HIV clinic (Continuity of care)
Schaufler et al. (2010)		Facilities support the screening of T2D	Prevention of T2D in subjects diagnosed with pre-diabetes by intensive lifestyle intervention	Support from health facility to healthcare providers to conduct preventive screening for diabetes	The use of diabetes screening guidelines		Prevention of T2DM in subjects diagnosed with pre-diabetes either by intensive lifestyle intervention or with metformin (Continuity of care)
Schouten et al. (2010)		Clinics supported the	Health care providers trained	Health care providers were directed and	Materials and information (change	System to register clinical parameters	The collaborative brings together and

		quality-improvement collaborative	on self-management support to patients	supported to change professional performance and care organisation and introduce patient self-management and a system to register clinical parameters.	package) about the structure of diabetes care, targets for glycaemic and cardiovascular risk control and therapy in a step-up regimen		supports multiprofessional diabetes teams from primary care and outpatient clinics (coordinated care)
Schuetz et al. (2013)*		Health care facilities/ organisations in the countries support the vascular disease health check interventions		The organisations and health care providers are supported appropriately to deliver the health checks to the population	Guidelines and standard operating procedures for health care providers exist and are used for the health checks		
Schultz et		MTM clinic	Pharmacists	Support for the		All visits are	Monthly face-to-face

al. (2021)		supports pharmacist-led services	provide adherence assistance for the patients	pharmacist-led activities in the MTM clinic		documented in the patient's electronic medical record, and medication changes are made in collaboration with the patient's providers.	visits to any patient in need of global medication and disease state assistance (Continuity of care) The clinic provides personalised and evidence-based medication regimen, adverse event monitoring, adherence assistance, and care coordination (person-centred care)
Wang et al. (2006)	Involvement of community social workers in the programme	Organisation supports the multiple disease screening		Support to health care providers to screen for breast, colorectal and liver cancers, cervical and oral	Use of screening guidelines		Coordination between healthcare providers participating in the Keelung programme

		programme		neoplasia, diabetes, hypertension, osteoporosis, and hyperlipidaemia			(Coordinated care) Referral to care for patients identified through the screening (Continuity of care)

Notes: MTM, medication therapy management; PEN, Package of essential NCD interventions; PN, practice nurse, GP, General practitioner; AF, atrial fibrillation; CKD, chronic kidney disease; BP, blood pressure; HIV, human immunodeficiency virus; ART, antiretroviral therapy; CDMP, chronic disease management program; T2D, type 2 diabetes; * The intervention was simulated and therefore assumptions made regarding the integrated care components

Supplementary table 5: Characteristics of the decision analytic models developed in the economic evaluations

Author (year)	Model type	Time horizon	Perspective	Discount rate	Costs and resource use parameters included	Disease parameters included	Analysis of sensitivity and uncertainty	Existing model adapted
Afzali et al. (2012)	SMM	40 years	Health care system	5%	- Patient-level data on hospital and primary care services	- History of diabetes<br>- History of comorbidities (e.g. PVD)<br>- History of diabetes-related complications.<br>- Time-varying risk factors (HbA1c, blood pressure, cholesterol level, smoking status)	- PSA (non- parametric bootstrapping)<br>- First-order uncertainty minimised by performing 1,000 repeated simulations per patient.	No
Dukpa et al. (2014)	Decision tree and a SMM	Lifetime	Societal	3%	- Screening<br>- Blood Pressure monitoring<br>- T2DM treatment and follow up<br>- HTN treatment and follow up	- Prevalence, proportions of HTN in T2DM patients<br>- Transitional probabilities - developing complications or death<br>- Sensitivity and specificity of screening tools	- PSA for parametric uncertainty<br>- First-order uncertainty (i.e., variability) was minimised by performing 1,000 repeated simulations per patient	No
Hirsch et al. (2017)	Individua l sampling model	2 years, 5 years, and 10 years	Health care system and payer	3%	- Intervention costs: Clinical pharmacists (including follow-up phone contact, nonpatient administrative cost, and patients time in the intervention clinic)<br>- Inpatient, outpatient, ambulatory, and treatment costs.	- Incidence, risk reduction of T2DM and related complications<br>- BMI, weight, blood pressure, cholesterol levels, triglycerides, fasting plasma glucose, HbA1c, and glomerular filtration rate.	- One-way and two-way sensitivity analysis, and PSA were conducted using upper and lower limits of 95% confidence intervals for clinical outcome parameters<br>- Probabilistic analyses simulated 1,000 replications	Archimedes model
Hobbs et al. (2005)	DES	Lifetime	Health system and patient	3.5%	- Screening costs<br>- Costs of stroke<br>- Costs of	- Prevalence & incidence of AF, first ischaemic stroke, Gastrointestinal bleed	- PSA was undertaken using distributions.<br>- A total of 10,000	No

			perspective		gastrointestinal bleed<br>- Cost of Warfarin treatment	- Rate, risks and probabilities of AF, stroke<br>- Relative risk, and risk reduction with medication.	replications was performed for 10,000 patients.	
Howard et al. (2010)	SMM	Lifetime	Health care funder perspective	5%	- Drug costs<br>- Out-patient health care utilisation (consultation costs for GP, dietician, podiatrist, nephrologist, diagnostic tests)	- Relative risks of CVD death<br>- CVD events<br>- Progression to microalbuminuria<br>- Progression to end-stage kidney disease for HTN and T2DM patients	- PSA was conducted	No
Kasaie et al. (2020)	Individua l sampling model	15 years	National and regional	3%	- Costs of standard care<br>- Costs of screening for hypertension and diabetes<br>- Costs of acute care for cardiac arrest, MI, angina, stroke, post-CHD, post-stroke<br>- Costs of HIV, hypertension, and diabetes treatment	- CVD natural history<br>- CVD Risk (10-year risk categories)<br>- Probability of first CHD event<br>- Acute and annual Mortality following CVD event<br>- NCD treatment effectiveness<br>- HIV, HTN, and diabetes prevalence<br>- ART coverage<br>- HIV incidence	- One-way sensitivity analysis was performed by varying the value of selected parameters to +/-15% of the original values	No
Kim et al. (2021)	SMM	Upto 60 years	Health care payer	5%	- Direct medical costs for hypertension, MI, stroke, CKD, heart failure<br>- Additional costs including pay-for-performance incentives to patients<br>- Costs for health support services.	- Incidence rates<br>- Mortality rates<br>- Effects of the chronic disease management program (Hazard ratios)	- One-way sensitivity analysis conducted based on probability, utility, and cost (Probabilities varied between a ±20% range, and cost up to 92.0 USD (100 000 KRW)).	No

Mason et al. (2005)	SMM	Lifetime	Health care system	5%	- Costs of drugs<br>- Costs for acute and chronic treatment after stroke and MI	- Risk of suffering a stroke or MI based on age, sex, and cardiovascular risk factors<br>- Risk of death for patients with stroke or MI by age and sex<br>- Mortality adjusted for diabetes<br>- Mortality from noncardiovascular causes<br>- Risk ratios for blood pressure and lipid lowering	- Uncertainty surrounding estimates explored using CEACs<br>- PSA conducted from Monte Carlo analyses with 10,000 evaluations of each model.	No
Mousa et al. (2021)	SMM	10-years	Public health provider	4.75%	- Medical procedure costs for CVD events<br>- Outpatient and inpatient costs<br>- Medication for outpatient visits<br>- Medication costs	- Changes in BP, HbA1c, total cholesterol, and HDL-cholesterol<br>- Risk of T2DM patients experiencing CVD events over 10 years	- PSA was conducted for the base-case analysis<br>- Monte-Carlo simulation with 10,000 iterations was performed to construct a CEAC.	No
Penaloza-Ramos et al. (2016)	SMM	Horizon varied from 30 years (lifetime) to between 1 - 20	UK NHS and Personal social services	3.5%	- Costs for ongoing BP monitoring in primary care, self-management, and prescription of antihypertensive agents<br>- Intervention costs: Self-management, equipment, and training costs.<br>- Replacement costs for equipment and training<br>- Costs for acute and chronic cardiovascular events.	- Blood pressure control<br>- Stroke risk<br>- Cardiovascular risk<br>- Mortality	- PSA was undertaken to incorporate parameter uncertainty, run with 10,000 second-order Monte Carlo simulations<br>- Cost-effectiveness planes and CEACs constructed to estimate the probability of self-management being cost-effective at different willingness-to-pay thresholds.	No
Sando et al. (2020)	Epidemio logic-cost	10 years	Health care system	3%	- Costs of medical consultation, laboratory,	- NCD risk factors<br>- Prevalence of HIV among	- Univariate sensitivity analyses, where varied one	No

	model				and medicines for hypertension, T2DM, and high cholesterol patients).<br>- Cost of hospitalisation for fatal or non-fatal CVD event and treatment costs	15–49-year-olds<br>- Proportion of HIV-infected enrolled in ART programs<br>- Prevalence of hypertension, T2DM, and hypercholesterolemia<br>- Proportion on medication for hypertension, DM, and high cholesterol<br>- Treatment efficacy for NCD risk factors	input parameter at a time independently while maintaining values for the other input parameters unchanged.	
Schaufler et al. (2010)	SPLMM	Lifetime	German statutory health insurance	Costs discounted at 5%<br>QALYs not discounted	- Costs for interventions: Screening test<br>- Verification of diagnosis costs<br>- Prevention using drugs and lifestyle interventions<br>- Costs for managing complications	- Age-dependent prevalence of pre-diabetes and T2DM<br>- Incidence of pre-diabetes and T2DM<br>- Incidence rates for complications<br>- Mortality rates<br>- Risk reductions due to prevention	- One-way sensitivity analysis on costs of early detection and prevention, discounting rates for costs and utilities, participation rates in the target population (5%, 15%, 45%, 30%), and effectiveness of early detection and prevention.	No
Schouten et al. (2010)	SMM	Lifetime	Health care system	Costs: 4.50 %<br>Effects: 1.50%	- Intervention costs: program-management costs<br>- Costs of the participating healthcare providers (time and related costs spent on the project, local overhead for each site, per patient annually).<br>- Health care delivery costs	- Life expectancy (Patient ageing)<br>- HRQoL<br>- Proportion of nonsmokers<br>- Proportion of patients with HbA1c <7 and <8.5%<br>- Hazard ratios for excess cardiovascular morbidity and mortality associated with diabetes<br>- Age-related risk of diabetes-specific complications	- One-way sensitivity analyses were conducted to test the robustness of the model results.<br>- PSA was performed to estimate the uncertainty surrounding the ICER.	No
Schuetz et al. (2013)	Individua l	30 years (Lifetime)	Health care system	3%	- Costs associated with the health check,	- Incidence and prevalence of T2DM and CVD events	- Not clearly reported	Archimedes model

	sampling model				subsequent testing and treatment, and cardiovascular events.	(MI, stroke, or CVD-related death)<br>- Incidence and prevalence of serious microvascular complications<br>- Medication use (e.g., anti-hypertensive, statin, and anti-diabetic treatments)<br>- Disease burden		
Schultz et al. (2021)	CTMC Model	10 years	Payer perspective	3%	- Costs of patient visits to intervention clinic<br>- Costs for primary prevention<br>- Costs of CVD events (states in the model including Stroke-tunnel, MI-tunnel, and other-tunnel states)	- Mean changes in HbA1c, blood pressure, emergency department and hospital admissions<br>- 10-year CVD risk<br>- Transition probabilities (Same CVD event, primary prevention to CVD event, recovery state of one CVD event to another CVD event)<br>- Mortality risk	- One-way sensitivity analysis (Risk ratios were varied by 10% above and below the base-case values, utilities by 20%, and costs by 50% to 200%).<br>- PSA were performed (A 10 000-iteration Monte Carlo simulation).<br>- CEACs were generated to display the Monte Carlo simulation results.	No
Wang et al. (2006)	SMM	20 years or 99 years (Lifetime)	Health care system	5%	- Screening costs<br>- Manpower cost<br>- Confirmation costs<br>- Costs for terminal care	- Disease progression<br>- Transition probabilities<br>- Natural history for each disease risk factor prevalence<br>- Survival<br>- Complication rates e.g., perforation in colonoscopy<br>- Association between non-malignant chronic diseases and cancers<br>- Screening performance parameters: Sensitivity and specificity	- Not reported	No

Notes: CTMC - continuous time Markov chain; DES - discrete event simulation; PSA- probabilistic sensitivity analysis; SMM - simulated markov model; SPLMM - simulated patient-level markov model; T2DM - type 2 diabetes mellitus; MI - myocardial infarction; PVD - peripheral vascular disease; ART - antiretroviral therapy; HIV - human immunodeficiency virus; BP - blood pressure; CEAC - cost-effectiveness acceptability curve; ICER - incremental cost-effectiveness ratio; CVD - cardiovascular disease; CKD - chronic kidney disease; HbA1c - glycated haemoglobin; QALYs - quality-adjusted life years; HRQoL - health-related quality of life; CHD - coronary heart disease

Supplementary note 3: Summary of results from the economic evaluations

Except for one, all the included studies found integrated care to be cost-effective at the chosen willingness-to-pay threshold. The resulting incremental cost effectiveness ratio (ICERs) in a study in Uganda were higher than the chosen WTP ($3,474), but the integration of NCD services into existing HIV care was associated with decreased 10-year CVD risk and lower ICERs among the older age groups (60 to 69 year-olds) [61]. In five studies conducted in Bhutan, USA, Korea, and UK, the analysis found that the integrated care intervention dominated the usual care alternative [62,63,73,78,82]. In all the included studies, the sensitivity analyses matched the base-case analysis where integrated care remained cost-effective at different modelling scenarios used. Results from one-way sensitivity analysis of the studies indicated that the parameters with the greatest impact on ICERs were, intervention/ treatment effectiveness inputs [61,63,76], intervention costs e.g. reimbursement of staff, treatment costs [61,76], health state utilities [85], screening/ treatment coverage [76], time horizon [77]. Cost-effectiveness acceptability curves (CEACs) were used in five studies [75,77,79,81,82] and showed cost-effectiveness of integrated care at a wide range of WTP thresholds.

Supplementary table 6: Results of the decision-analytic models in the economic evaluations

		Costs and resource use			Outcomes/ benefits			Inc. Costs	Inc. Outcome	CE threshold	ICER (Base Case)	Conclusion
**Author (year)**	**Interventions**	**Currency**	**Intervention**	**Comparator**	**Measure**	**Intervention**	**Comparator**					
Afzali et al. (2012)	High-level PN involvement	AUD ($)	$56,779	$65,517	QALYs	6.8 (6.3–7.3)	6.5 (5.9–7.0)	-$8,738 (-$12,522 to -$4,954)	0.3 (0.2–0.4)	Not reported	Not reported	Cost-effective
Dukpa et al. (2014)	PEN program	BTN	BTN 205 735	BTN 210 023	DALYs averted	-	-	-	0.038	159 168- 477 504 BTN/DALY averted	-112 906	Very cost-effective
	Universal screening	BTN	BTN 203 897	BTN 210 024	DALYs averted	-	-	-	0.016	160 168- 477 504 BTN/DALY averted	-112 907	Very cost-effective
Hirsch et al. (2017)	DIMM clinic (2 years)	USD ($)	$899,371	$962,565	QALYs	97	96	-	-	Not reported	-63,194/QALY	Cost-effective
	DIMM clinic (5 years)	USD ($)	$2,137,659	$2,272,572	QALYs	222	218	-	-	Not reported	-33,728/QALY	Cost-effective
	DIMM clinic (10 years)	USD ($)	$3,879,964	$4,114,363	QALYs	385	375	-	-	Not reported	-23,440/QALY	Cost-effective
Hobbs et al. (2005)	Opportunistic screening	GBP (£)	Not reported	Not reported	Cases detected	75 (59–94)	47 (35–62)	£10,174 (£9593 to £10,755)	28	Not reported	363/ case detected	Cost-effective

	Systematic high risk	GBP (£)	Not reported	Not reported	Cases detected	53 (40–69)	48 (35–62)	£24,530 (£23,678 to £25,382)	6	Not reported	Dominated by opportunistic screening	Systematic high risk: Dominated by opportunistic screening
	Systematic population	GBP (£)	Not reported	Not reported	Cases detected	74 (58–93)	49 (35–62)	£48,260 (£46,952 to £49,567)	27	Not reported	Dominated by opportunistic screening	Systematic population: Dominated by opportunistic screening
Howard et al. (2010)	Primary care screening plus intensive treatment of diabetes	AUD ($)	$17,832 ($3,027–70,025	$16,487 ($1,875–68,20	QALYs	12.798 (4.321–17.720)	12.701 (4.144–17.627	$1,345 (-$6,600–9,902)	0.097 (–0.408–0.696	$A50,000	$13,866/QALY	Cost-effective
	Primary care screening plus intensive treatment of hypertension	AUD ($)	$14,061 ($1,178–61,009)	$14,004 ($1,402–63,661)	QALYs	12.947 (4.768–18.037	12.831 (4.673–17.69	$57 (-$8,058–7,75	0.116 (–1.396–1.745)	$A50,000	$491/QALY	Cost-effective
	Primary care screening plus intensive treatment of protenuria	AUD ($)	$16,974 ($1,867–65,23	$16,821 ($1,641–64,826	QALYs	12.763 (4.871–17.806)	12.731 (4.828–17.806)	$153 (-$7,708–7,5	0.032 (–0.790–0.9	$A50,000	$4,781/QALY	Cost-effective

Kasaie et al. (2020)	Community-based integrated HIV-NCD screening and treatment	USD ($)	860.36 [830.59 to 890.66]	-	DALYs averted	-	-	6.68 [6.61 to 6.74] billion	7.76 [8.01 to 7.51] million	USD 2,010	$860.30 per DALY averted.	Cost-effective
Kim et al. (2021)	CDMP	Korean Won (KRW)	CDMP: 32 774 249	Usual care: 35 473 613	QALYs	16.6	16.1	Incremental: 2 699 364	Incremental: -0.46855	USD 33 429	−5 761 088 (Usual care dominated by CDMP)	Highly Cost-effective
Mason et al. (2005)	Nurse-led clinics (BP control)	USD ($)	$306,400	Not reported	QALYs	0.53/patient	Not reported	-	-	$50,000/QALY	−$1,400/QALY	Cost-effective
	Nurse-led clinics (Lipid lowering)	USD ($)	$306,400	Not reported	QALYs	0.46/patient	Not reported	-	-	$50,000/QALY	$8,230/QALY	Cost-effective
Mousa et al. (2021)	Pharmacist-led care	JD	6668.5 (6244.29,7687.03)	5391.2 (4931.6,6556.9)	Life years gained (LYG)	4.2 (4.1,4.3)	3.9 (3.8,4.0)	1238.78	0.3 LYG/patient	(JD3,008.36) US$4,241.79 (very cost-effective) to (JD9,023.23) US$12,723 (cost-effective)	JD4058.5 (3208.8,6117.8) per LY gained	Cost-effective

Penaloza-Ramos et al. (2016)	Self-management	GBP (£)	7357	8187	QALYs	6.2466	6.0326	–830	0.2139	£20,000/QALY gained	Self-management is Dominant	Cost-effective
Sando et al. (2020)	Integrated screeening and treatment (Women 60-69 years)	USD ($)	$10,541,000	-	DALYs averted	7305<br>-	-	-	-	$3,474	1445/ DALY averted	Cost-effective among older age-groups<br>Not cost-effective among 30-44 years age group
	Integrated screeening and treatment (Men 60-69 years)	USD ($)	$2386000	-	DALYs averted	1700	-	-	-	$3,474	1400/DALY averted	Cost-effective among older age-groups<br>Not cost-effective among 30-44 years age group
Schaufler et al. (2010)	Lifestyle intervention	Euro (€)	24700	23000	QALYs	-	-	€1637	2.91	Not reported	€562.54 per QALY	Cost-effective in the longterm
	Prevention with metformin	Euro (€)	24000	23000	QALYs	-	-	€ 921	2.83	Not reported	€325.44 per QALY	Cost-effective in the longterm
Schouten et al. (2010)	Quality improvement collaborative	Euro (€)	-	-	QALYs	-	-	€ 860	0.33	€20,000 to €80,000 per QALY gained	2570/discounted QALY	Cost-effective

	(Men)											
	Quality improvement collaborative (Women)	Euro (€)	-	-	QALYs	-	-	€ 643	0.26	€20,000 to €80,000 per QALY gained	€2448/ discounted QALY	Cost-effective
Schuetz et al. (2013)	Standardized vascular disease health check	Euro (€)	Country specific costs not reported	Country specific costs not reported	QALYs	Country specific QALYs not reported.	Country specific QALYs not reported.	-	-		Cost per QALY gained (ICER) Denmark: 11595 France: 14903 Germany: 115 Italy: 11113 Poland: Cost saving UK: 2426	Cost-effective in all study countries
Schultz et al. (2021)	MTM program	USD ($)	$1 378 052	0	QALYs	1402	1384	$1 378 052	18	$100,000	$38 798/QALY	Cost-effective
Wang et al. (2006)	Multiple disease screening (100%)	USD ($)	154,139,60 8	151,290,56 2	Life years gained (LYG)	854,143.59	849,871.29	4,272.30	-	Not reported	US$667	Multiple screening may be more cost-effective than single disease screening

Notes: Base case results from selected studies are presented.

Supplementary table 7: Quality assessment of included studies

Dimension of quality			a	b	c	d	e	f	g	h	i	j	k	l	m	n	o	p	Score (%)
**Structure**		**Item #**																	
S1	Statement of decision problem/ objective	1	Y	Y	Y	Y	Y	Y	Y	Y	Y	Y	Y	Y	Y	Y	Y	Y	100.0
		2	Y	Y	Y	Y	Y	Y	Y	Y	Y	Y	Y	Y	Y	Y	Y	Y	100.0
		3	U	Y	U	U	U	U	Y	X	U	Y	U	Y	U	X	U	X	53.1
S2	Statement of scope/ perspective	1	Y	Y	Y	Y	Y	Y	Y	X	Y	Y	Y	Y	Y	U	Y	X	84.4
		2	Y	Y	Y	Y	Y	Y	Y	Y	Y	Y	Y	Y	Y	U	Y	U	93.8
		3	Y	U	Y	Y	Y	Y	Y	Y	Y	Y	Y	Y	Y	Y	Y	U	93.8
		4	Y	Y	Y	U	Y	Y	Y	Y	Y	Y	Y	Y	Y	Y	Y	U	93.8
S3	Rationale for structure	1	Y	Y	Y	Y	Y	Y	Y	Y	Y	Y	Y	Y	Y	Y	Y	U	96.9
		2	Y	Y	Y	Y	Y	Y	Y	Y	Y	Y	Y	Y	Y	Y	Y	Y	100.0
		3	U	Y	U	Y	Y	Y	Y	Y	Y	Y	Y	Y	U	Y	Y	U	87.5
S4	Structural assumptions	1	Y	Y	Y	Y	Y	Y	Y	Y	Y	Y	Y	Y	Y	Y	Y	Y	100.0
		2	Y	Y	Y	Y	Y	Y	Y	Y	Y	Y	Y	Y	Y	Y	Y	Y	100.0
S5	Strategies/ comparators	1	Y	Y	Y	Y	Y	Y	Y	Y	Y	Y	Y	Y	Y	Y	Y	Y	100.0
		2	U	Y	U	U	U	U	U	U	U	U	Y	Y	U	U	X	Y	59.4
		3	U	N/A	Y	U	N/A	N/A	U	U	U	U	N/A	U	X	U	X	N/A	45.5
S6	Model type	1	Y	Y	Y	Y	Y	Y	Y	Y	Y	Y	Y	U	X	Y	Y	Y	90.6
S7	Time horizon	1	Y	Y	Y	Y	Y	Y	Y	Y	Y	Y	Y	Y	Y	Y	Y	Y	100.0
		2	Y	Y	Y	Y	Y	Y	Y	Y	Y	Y	Y	Y	Y	Y	Y	Y	100.0
S8	Disease states/ pathways	1	Y	Y	Y	Y	Y	Y	Y	Y	Y	Y	Y	Y	Y	U	Y	U	93.8
S9	Cycle length	1	Y	Y	Y	Y	Y	Y	Y	Y	Y	Y	X	Y	X	N/A	Y	X	80.0
**Data**																			
D1	Data identification	1	Y	Y	Y	Y	U	Y	Y	Y	Y	Y	Y	Y	Y	Y	Y	Y	96.9
		2	Y	Y	Y	Y	Y	Y	Y	Y	Y	Y	Y	Y	Y	Y	Y	Y	53.1

		3	Y	Y	U	Y	Y	Y	Y	Y	Y	Y	Y	Y	Y	Y	Y	Y	64.3
		4	U	U	U	Y	U	U	U	U	U	U	U	U	U	Y	U	X	96.9
		5	N/A	U	U	Y	N/A	U	U	U	Y	U	Y	U	U	Y	U	U	100.0
D2	Data modelling	1	Y	Y	Y	Y	Y	Y	Y	Y	Y	Y	Y	Y	Y	Y	Y	U	96.9
D2a	Baseline data	1	Y	Y	Y	Y	Y	Y	Y	X	Y	Y	Y	Y	Y	Y	Y	Y	53.1
		2	Y	Y	Y	U	U	Y	Y	Y	Y	Y	Y	Y	U	Y	Y	U	64.3
		3	U	U	U	U	U	U	U	U	Y	U	U	U	U	N/A	X	U	96.9
		4	U	U	U	U	U	U	U	U	N/A	U	U	U	U	N/A	X	U	93.8
D2b	Treatment effects	1	Y	N/A	Y	Y	Y	Y	U	N/A	N/A	N/A	N/A	N/A	Y	Y	Y	N/A	87.5
		2	Y	Y	Y	Y	Y	Y	U	X	Y	Y	Y	X	Y	Y	Y	U	50.0
		3	Y	Y	Y	Y	Y	Y	Y	Y	Y	Y	Y	Y	Y	Y	Y	U	46.4
		4	Y	N/A	Y	U	U	Y	U	X	X	Y	X	U	X	X	X	U	94.4
		5	Y	Y	Y	Y	Y	Y	Y	Y	Y	Y	Y	Y	Y	Y	Y	U	81.3
D2c	Costs	1	Y	Y	Y	Y	Y	Y	Y	Y	Y	Y	Y	Y	Y	Y	Y	Y	96.9
		2	Y	Y	Y	Y	Y	Y	Y	Y	Y	Y	Y	Y	Y	Y	Y	X	43.3
		3	Y	Y	Y	Y	Y	Y	Y	Y	Y	Y	Y	Y	Y	Y	Y	Y	100.0
D2d	Quality of life weights (utilities)	1	Y	Y	Y	Y	Y	Y	Y	Y	Y	Y	Y	Y	Y	Y	Y	Y	100.0
		2	Y	Y	X	Y	Y	X	Y	Y	Y	Y	Y	Y	Y	Y	Y	X	81.3
		3	Y	Y	Y	U	X	U	U	Y	Y	Y	Y	Y	Y	Y	Y	X	78.1
D3	Data incorporation	1	Y	Y	Y	Y	Y	Y	Y	Y	Y	Y	Y	Y	Y	Y	Y	Y	100.0
		2	Y	Y	U	U	U	Y	Y	Y	Y	Y	Y	U	X	U	U	Y	75.0
		3	Y	Y	Y	Y	Y	Y	Y	U	Y	Y	Y	Y	Y	Y	Y	U	93.8
		4	U	Y	X	Y	Y	U	U	Y	Y	Y	X	X	U	X	U	U	56.3
		5	Y	Y	U	Y	Y	U	X	Y	Y	Y	X	U	U	U	X	U	62.5
D4	Assessment of uncertainty	1	X	X	X	Y	X	X	X	U	X	Y	X	X	X	X	X	X	15.6
		2	X	X	X	N/A	X	X	X	U	U	N/A	X	X	X	X	X	X	7.1
D4a	Methodological	1	Y	Y	Y	Y	Y	Y	X	X	X	Y	U	X	X	X	X	X	46.9
D4b	Structural	1	Y	Y	Y	Y	Y	Y	Y	Y	Y	Y	Y	Y	Y	Y	Y	X	93.8

D4c	Heterogeneity	1	X	X	X	Y	X	Y	X	X	X	Y	Y	Y	X	X	X	X	31.3
D4d	Parameter	1	Y	Y	Y	Y	Y	Y	Y	Y	Y	Y	Y	Y	U	Y	Y	U	93.8
		2	Y	Y	Y	Y	Y	Y	Y	Y	Y	Y	U	Y	X	Y	Y	U	87.5
**Consistency**																			
C1	Internal consistency	1	Y	U	Y	Y	U	U	X	U	U	U	U	U	Y	Y	U	X	59.4
C2	External consistency	1	Y	N/A	Y	Y	N/A	Y	N/A	Y	Y	Y	Y	Y	U	Y	N/A	Y	95.8
		2	N/A	N/A	N/A	U	U	Y	N/A	Y	U	U	U	U	U	Y	U	Y	66.7
		3	Y	Y	Y	Y	Y	Y	Y	Y	Y	Y	Y	Y	Y	Y	X	X	87.5
**Overall Score**																			80.8

Y: Yes, X: No, N/A: Not applicable, U: Unclear; a =Afzali *et al.* (2012), b = Dukpa *et al.* (2014), c = Hirsch *et al.* (2017), d = Hobbs *et al.* (2005), e = Howard *et al.* (2010), f = Kasaie *et al.* (2020), g = Kim *et al.* (2021), h = Mason *et al.* (2005), i = Mousa *et al.* (2021), j = Ramos *et al.* (2016), k = Sando *et al.* (2020), l = Schaufler *et al.* (2010), m = Schouten *et al.* (2010), n = Schuetz *et al.* (2013), o = Schultz *et al.* (2016), p = Wang *et al.* (2006)
